# Glycemic Index, Glycemic Load, and FAS rs6586161 Polymorphism in Relation to Gastric Cancer Risk: A Case-Control Study in Korea

**DOI:** 10.3390/nu15143238

**Published:** 2023-07-21

**Authors:** Hong Kyoung Kim, Sang Young Kim, Jung Hyun Kwak, Hyun Ja Kim

**Affiliations:** 1 Department of Acupuncture and Moxibustion Medicine, College of Korean Medicine, Daejeon University, 62, Daehak-ro, Dong-gu, Daejeon 34520, Republic of Korea; goheekoo@naver.com; 2 Department of Food and Nutrition, Gangneung-Wonju National University College of Life Science, 7 Jukheon-gil, Gangneung-si 25457, Republic of Korea; sangaustin@gmail.com (S.Y.K.); hyun4615@hanmail.net (J.H.K.)

**Keywords:** case-control study, gastric cancer, glycemic index, glycemic load, single-nucleotide polymorphisms, FAS, rs6586161

## Abstract

Many dietary and genetic factors have been confirmed to be associated with gastric cancer risk. This research investigated gastric cancer risk with regard to the glycemic index, glycemic load, and FAS rs6586161 polymorphism. A total of 232 matched pairs were included in this case-control study. Data collection was conducted at two hospitals in Korea from 2002 to 2006. Dietary information was obtained from a food frequency questionnaire, and genotypes of FAS rs6586161 polymorphism were TT, TA, and AA type. Gastric cancer risk was increased for the highest tertile of glycemic index (vs. lowest tertile, OR = 1.84, 95% CI = 1.07–3.18), the highest tertile of glycemic load (vs. lowest tertile, OR = 2.14, 95% CI = 1.23–3.75), and the AA type of FAS rs6586161 polymorphism (vs. TT types, OR = 1.95, 95% CI = 1.13–3.39). Furthermore, gastric cancer risk was significantly elevated for the participants with the highest glycemic load and AA type of FAS rs6586161 polymorphism (vs. the lowest glycemic load and TT type, OR = 5.53, 95% CI = 2.01–15.21). Both the high glycemic load and AA type of FAS rs6586161 polymorphism increased gastric cancer risk; however, the interactions between these two elevated the risk of gastric cancer even more.

## 1. Introduction

Based on the Global Cancer Statistics 2020, gastric cancer ranked fifth in incidence and fourth in mortality [1]. Korea had the third highest gastric cancer rate after Mongolia and Japan [2]. In previous studies, many risk factors for gastric cancer have been verified, for example, genetics, *Helicobacter pylori* (*H. pylori*) infection, smoking, alcohol drinking, and diet [3]. In particular, with regard to dietary factors, carbohydrate consumption needs to be carefully monitored given that the main source of energy is carbohydrates in Korea. Specifically, energy intake from carbohydrates contributed 59.5% of the total energy intake for Korean adults, followed by 16.3% of protein and 24.3% of fat, according to the 2021 Korea National Health and Nutrition Examination Survey [4]. In addition to assessing carbohydrate consumption itself, it is important to consider the glycemic index, which indicates the quality of carbohydrate intake. The glycemic index is a numerical value of how quickly blood glucose levels increase after consuming carbohydrate foods, and glycemic load further considers the amount of carbohydrates consumption [5]. A diet with a high glycemic index or glycemic load can consistently raise glucose levels. A chronic high glucose level can promote cancer development by hyperglycemia and hyperinsulinemia, which are related to elevated risk of tumor growth [6]. In our previous study, we found that gastric cancer risk was increased with a high glycemic index and glycemic load, respectively [7].

Tumor development can be caused by factors such as the activation of oncogenes or the reduction of tumor suppressors [8]. As a tumor suppressor, the FAS pathway plays an important role. When DNA somatic transformation of this pathway occurs, it loses its function as a suppressor, resulting in increased cancer incidence as the pathway controls cell proliferation and apoptosis of inappropriate cells [9,10]. As one of the epigenetic effects, DNA methylation of various cancer-related genes, such as FAS, can be caused by inappropriate glucose metabolism [11]. To analyze the above mechanism, we tried to identify the association with the rs6586161 gene, which had a clear association with gastric cancer among the single-nucleotide polymorphisms (SNPs) of the FAS gene [12]. In addition, a diet with high glycemic index and glycemic load levels induces inactivation of the FAS pathway, which regulates cell growth, proliferation, migration, differentiation, and apoptosis, leading to tumor growth [13].

Each dietary and genetic factor is a known risk factor for gastric cancer, however, studies on gastric cancer considering both factors simultaneously are scarce in Korea. Therefore, the purpose of this case-control study was to investigate the association between FAS rs6586161 polymorphism, carbohydrate consumption, glycemic index, and glycemic load with gastric cancer risk. Furthermore, we examined how the interacted association of glycemic load, which considered both quality and quantity of carbohydrate consumption, and FAS rs6586161 polymorphism affected gastric cancer risk.

## 2. Materials and Methods

### 2.1. Study Design and Participants

This was a case-control study, and data collection was performed at the Chungnam University Hospital and the Hanyang University Guri Hospital. A total of 925 participants (440 cases and 485 controls) were collected, and the age range of the participants was from 20 to 79 years. Given the slight alteration of the survey questionnaires, there were two different time points, the first stage of December 2002–August 2003 and the second stage of October 2003–September 2006. To diagnose gastric cancer, a gastroenterologist performed a gastroscopy, and then gastric cancer was confirmed by a pathologist via a histopathologic examination. Individuals in the control group were recruited among patients who visited one of the following departments: orthopedics, otolaryngology, neurosurgery, ophthalmology, thoracic surgery, urology, neuropsychiatry, and dermatology. A gastroscopy was used to confirm the absence of gastric cancer. Individuals with energy consumption <500 kcal or >5000 kcal were excluded. The final 232 pairs of cases and controls were selected through a 1:1 matching procedure considering sex, age of ±5 years, participating hospital, and participation period of ±1 year. Written informed consent was acquired from the participants, and the research procedure was overseen by the Institutional Review Board (IRB) of Hanyang University Medical Center (no. 2003-4).

### 2.2. Data Collection

Data collection was completed by well-disciplined nurses, including sociodemographic, behavioral, clinical, and dietary characteristics. There were four groups according to body mass index as underweight (<18.5 kg/m^2^), normal (18.5–22.9 kg/m^2^), overweight (23.0–24.9 kg/m^2^), or obese (≥25.0 kg/m^2^), and four groups according to education level as ≤elementary school, middle school, high school, or ≥college. Smoking status was classified as never, past, or current, and alcohol drinking status was divided into never, past, <20 g/day for women or <40 g/day for men, or ≥20 g/day for women or ≥40 g/day for men. Furthermore, a rapid urea degradation test using Campylobacter-like organism test kit (Kimberly-Clark/Ballard Medical Products, Draper, UT, USA; product no. 60480) was performed to assess *H. pylori* infection. For dietary intake, a food frequency questionnaire (FFQ) was used, which was from the slightly modified version of the validated FFQ in the past research [14]. As participants with gastric cancer may have modified their diet, the FFQ was conducted based on recalling food consumption for 1 year of the past 3 years to assess remote dietary consumption. The FFQ consisted of 102 foods or dishes in the first stage and 115 foods or dishes in the second stage. In addition, the average serving size was listed in both stages. The daily amount of each food item was calculated using the intake frequency and serving size. In more detail, it was obtained by multiplying the frequency value, converted to the daily frequency, by the average serving size. A specific explanation regarding the dietary data collection has been presented in the past study [7]. Calculation of total energy and carbohydrate consumption was performed using the Computer-Aided Nutritional Analysis program by the Korean Nutrition Society [15]. A total glycemic index value was calculated by multiplying the glycemic index of each food item by the carbohydrate content of the food. Then, the sum of these values was divided by the total carbohydrate intake. The glycemic index value of each food item was derived from the information provided by Foster-Powell et al., (2002) [16], Atkinson et al., (2008) [17], and Kyung Hee University (2015) [18]. In order to obtain glycemic load, the carbohydrate content of each food was multiplied by its glycemic index, then all the values were added and divided by 100.

### 2.3. SNP Genotyping

For SNP, FAS rs6586161 polymorphism was selected based on the review of genes related to gastric cancer, carbohydrates, glycemic index, and glycemic load [12]. For the collection process of the SNP, firstly, peripheral blood leukocytes were isolated from the whole blood to extract genomic DNA. Then, the primary quality control of DNA was completed via a NanoDrop ND-1000 spectrophotometer (Thermo Fisher Scientific Inc., Wilmington, NC, USA). Moreover, the KASP assay [19] was conducted to define the SNP genotype through a QuantStudio 5 Real-Time PCR Instrument (Thermo Fisher Scientific Inc., Wilmington, NC, USA). Finally, there were three genotypes of FAS rs6586161 polymorphism: TT, TA, and AA. This present study had no genotyping error by passing the criteria of the minor allele frequency (MAF) of >1%, *p*-value for Hardy–Weinberg equilibrium (HWE) of >0.05, and SNP call rate of >95%.

### 2.4. Statistical Analyses

SAS software version 9.4 (SAS Institute, Cary, NC, USA) was used for all the statistical analyses. The general characteristics of the participants are presented by cases and controls. Continuous variables and categorical variables are shown as mean ± standard deviation (SD) and numbers (percentages), respectively. The *t*-test and Chi-square test were conducted to compare cases and controls. Moreover, each carbohydrate consumption, glycemic index, and glycemic load were grouped as tertiles—the lowest, medium, and highest groups. In the process of grouping, the two different stages of recruitment were considered. For FAS rs6586161 polymorphism, three models were considered for the analyses: a model consisting of the TT, TA, and AA types; a dominant model of the TT and TA+AA types; and a recessive model of the TT+TA and AA types. In order to calculate odds ratios (ORs) and 95% confidence intervals (95% CIs) of gastric cancer, multivariable logistic regression was used, controlling for general gastric cancer risk factors. Specifically, covariates of the first model were sex (men or women), age (years, continuous), body mass index (<18.5, 18.5–22.9, 23.0–24.9, ≥25.0 kg/m^2^, or missing), family history of gastric cancer (no or yes), hospital (Chungnam National University or Hanyang University Guri), education (≤elementary school, middle school, high school, ≥college, or missing), smoking (never, past, or current), alcohol drinking (never, past, <20 g/day for women or <40 g/day for men, or ≥20 g/day for women or ≥40 g/day for men), and *H. pylori* infection (negative, positive, or missing). Additionally, the second model was further controlled for fruit intake (g/day, continuous), vegetable intake (g/day, continuous), and non-carbohydrate energy intake (g/day, continuous). Moreover, a likelihood ratio test was completed to examine the interactions between glycemic load and FAS rs6586161 polymorphism; specifically, the logistic regression model included a multiplicative interaction term of glycemic load and FAS rs6586161 polymorphism. Statistical significance was defined as *p*-values of <0.05.

## 3. Results

### 3.1. General Characteristics

Table 1 describes the general characteristics of cases of gastric cancer and controls. The 232 matched pairs of cases and controls included 158 men (68.1%) and 74 women (31.9%) in each group. The average age of cases and controls was 57.2 ± 11.8 and 56.6 ± 11.6 years, respectively. There was a higher proportion of obesity group for controls (32.3%) in comparison with cases (22.4%) (*p* = 0.027). No differences were observed between cases and controls for family history of gastric cancer, education, and smoking. For alcohol drinking, the percentage of the participants with ≥20 g/day for women or ≥40 g/day for men was greater in cases (24.1%) compared to the controls (17.7%) (*p* = 0.002). Additionally, the percentage of the participants with *H. pylori* infection for controls (45.3%) was higher than cases (30.6%) (*p* = 0.003). For FAS rs6586161 polymorphism, a greater proportion of the participants were in the AA type in cases (24.6%) than controls (16.4%) (*p* = 0.043). Also, cases compared to controls had significantly higher total energy intake (1854.4 kcal/day ± 713.9 vs. 1703.5 kcal/day ± 593.4, *p* = 0.014) and glycemic load (181.2 ± 68.9 vs. 163.2 ± 50.9, *p* = 0.002), unlike glycemic index (58.5 ± 3.4 vs. 58.4 ± 3.1, *p* = 0.597).

### 3.2. Gastric Cancer Risk by Gastric Cancer Risk Factors

Table 2 illustrates the risks of gastric cancer according to general risk factors for gastric cancer, controlling for confounding factors. An increased risk of gastric cancer was observed for the past drinkers (OR = 2.15, 95% CI = 1.09–4.23), while a decreased risk was shown for the participants with *H. pylori* infection (OR = 0.47, 95% CI = 0.29–0.77). However, there were no significant associations between gastric cancer risk and other risk factors of body mass index, family history of gastric cancer, education, and smoking.

### 3.3. Gastric Cancer Risk by Carbohydrate Intake, Glycemic Index, Glycemic Load, and FAS rs6586161 Polymorphism

Table 3 shows the risks of gastric cancer with regard to carbohydrate intake, glycemic index, glycemic load, and FAS rs6586161 polymorphism. There was no relationship between gastric cancer risk and carbohydrate consumption. However, when comparing the highest tertile to the lowest tertile, an elevated risk of gastric cancer was noticed for glycemic index (OR = 1.84, 95% CI = 1.07–3.18) and glycemic load (OR = 2.14, 95% CI = 1.23–3.75). For FAS rs6586161 polymorphism, increased gastric cancer risk was observed for the AA type compared to the TT type (OR = 1.95, 95% CI = 1.13–3.39). Similarly, for the recessive model, the AA type had an elevated risk of gastric cancer in comparison with the TT+TA type (OR = 1.77, 95% CI = 1.08–2.89), unlike the dominant model.

### 3.4. Gastric Cancer Risk by Interactions of Glycemic Load and FAS rs6586161 Polymorphism

Table 4 presents gastric cancer risks related to the interactions of glycemic load and FAS rs6586161 polymorphism. For the individual types of FAS rs6586161 polymorphism, there was a significant association with increased gastric cancer risk for the highest glycemic load and AA type of FAS rs6586161 polymorphism in comparison with the lowest glycemic load and TT type of FAS rs6586161 polymorphism (OR = 5.53, 95% CI = 2.01–15.21). (However, for glycemic index, increased gastric cancer risk was observed for the participants with high glycemic index regardless of the FAS rs6586161 polymorphism types (OR = 3.73, 95% CI = 1.52–9.14 for TT type, OR = 2.62, 95% CI = 1.14–6.01 for TA type, and OR = 3.77, 95% CI = 1.46–9.76 for AA type, data not shown). For the dominant model, classified as TT and TA+AA types, gastric cancer risk was elevated for the highest glycemic load and TA+AA type compared to the lowest glycemic load and TT type (OR = 2.94, 95% CI = 1.31–6.60). In addition, for the recessive model of TT+TA and AA types, an increased gastric cancer risk was revealed for participants who belonged to the highest glycemic load group and had the TT+TA type (OR = 2.05, 95% CI = 1.11–3.76) or AA type (OR = 5.00, 95% CI = 2.08–11.99) as compared to those of the lowest glycemic load and TT+TA type. The interaction between glycemic load and individual types of FAS rs6586161 polymorphism was significant (*p* < 0.05). However, no interaction was found for glycemic load with the dominant and recessive model of FAS rs6586161 polymorphism, respectively.

## 4. Discussion

This case-control study found that each of a high glycemic index, high glycemic load, and FAS rs6586161 polymorphism of AA type increased gastric cancer risk. Furthermore, the risk of gastric cancer was significantly increased for participants with the interaction of the highest glycemic load and AA type of FAS rs6586161 polymorphism compared to participants with the lowest glycemic load and TT type of FAS rs6586161 polymorphism.

There are many patients suffering from gastric cancer, and the rate of gastric cancer is high in Korea (third highest worldwide) [2]. Accordingly, management of the disease through analysis of gastric cancer-related factors is important. Carbohydrates are a major source of energy for Koreans; however, chronically increasing blood sugar levels via high carbohydrate intake can cause various chronic diseases [20]. The association between a carbohydrate diet and the incidence of gastric cancer was examined by previous studies [7,21]. Specifically, it was reported that a diet with high glycemic index and glycemic load levels increases the incidence of gastric cancer. In this present study, there were also significant associations between glycemic index and glycemic load with gastric cancer. Glycemic index is a value related to the type of carbohydrate consumed, and glycemic load is a value that considers both the type and amount of carbohydrates at the same time [21]. Food intake with high glycemic index and glycemic load levels increases postprandial blood glucose, which can chronically increase insulin levels that activate the insulin-like growth factor (IGF) system. Hyperinsulinemia appears when the regulating function of glucose load is impaired. As it inhibits apoptosis by activating IGF, the proliferation of cells related to gastric cancer occurs. The expression of IGF was shown in the cell lines of gastric cancer patients, and a higher level was observed than that of controls [22]. Previous studies have shown that high glycemic index and glycemic load levels increase the incidence of gastric, colorectal, breast, endometrium, ovary, and bladder cancers and type 2 diabetes [23,24]. It suggests that glycemic index and glycemic load levels are highly associated with the risk of gastric cancer, and the quality of the diet is more important than the total amount of carbohydrates in the diet. Furthermore, it is necessary to monitor closely for glycemic load more than glycemic index, given that it is important to consider how much individuals consume the food as well, in addition to the quality of the food itself. Each food has its glycemic index and glycemic load, and it is expected that gastric cancer can be prevented through a slowly absorbed carbohydrate diet.

Given that apoptosis is a process that genetically regulates various cells that exhibit inappropriate conditions, it plays a major role in maintaining metabolic homeostasis [25]. As it is also involved in the remodeling of cellular constituents of the vessel wall, it was reported that this process is closely related to vascular diseases such as hypertension and diabetes [26,27]. Among the various apoptotic pathways, FAS, which is a cell surface receptor that belongs to the tumor necrosis factor receptor family, is primarily involved in the process of apoptosis [28]. It plays a role in controlling tumor progression by transducing apoptotic signals to various organs through FAS-mediated apoptosis. In other words, given that the inactivation of this pathway leads to carcinogenesis, it was reported that FAS expression is mainly attributable to patient survival [29,30]. According to previously reported studies, the FAS pathway was reported to be closely related to diseases [31,32].

Many studies have been conducted to establish a database of SNPs to identify the occurrence of disease in advance. These studies are expected to help prevent or detect diseases clinically [33]. According to previous studies, rs6586161, one of the SNPs involved in the FAS pathway, was reported to cause gastric cancer by showing an association with the AKT signaling pathway [12,34,35]. However, existing studies related to the clear identification of the mechanism are insufficient.

As a result of comparing the association with the FAS rs6586161 polymorphism and gastric cancer risk, the AA type had a higher gastric cancer risk than the TT type or TT+TA type in the recessive model. The AA type, corresponding to the minor allele, causes problems with the function of FAS-mediated apoptosis, which increases the incidence of gastric cancer by failing to inhibit the activation of gastric cancer cells. As a high-glycemic-load diet can influence inactivation of the FAS pathway, the combined effects of the AA type and a high glycemic load can lead to negative synergy, significantly increasing the risk of gastric cancer. Thus, individuals with the AA type and a high glycemic load can have significantly elevated gastric cancer risk, which was confirmed by our findings.

Several strengths exist in this present study. Research on the interactions between glycemic load and FAS rs6586161 polymorphism in relation to the risk of gastric cancer has not been previously published, to the best of our knowledge. Next, interviews with participants were completed without being informed about the status of their disease after endoscopic examination to reduce information bias. Moreover, the recruitment of the control group was completed during the same period at the same hospital to decrease misclassification bias.

Yet, there are various limitations. It is difficult to describe the participants as representative of the Korean population because data were collected at two hospitals. Additionally, the slight difference in FFQ items between the first and second stages could be a limitation. Also, it is possible that recall bias occurred because participants were asked to recall their diet for 1 year of the previous 3 years. However, acceptable reproducibility and validity of the FFQ collected at 3-year intervals were reported [36]. Furthermore, the controls had a greater infection rate of *H. pylori* compared to the cases. This could be due to several reasons, including antibiotic treatment resulting in the absence of *H. pylori* and low *H. pylori* detection according to gastric carcinogenesis progression. In addition, residual confounding effects may have remained even though various confounding factors were controlled. Finally, there might be more SNPs available in addition to the particular SNP studied, the FAS rs6586161 polymorphism. More SNPs related to both glycemic load and gastric cancer are needed to explore in future studies.

Conclusively, the risk of gastric cancer was increased for patients with a high glycemic index, high glycemic load, and the AA type of FAS rs6586161 polymorphism, respectively. Moreover, gastric cancer risk was more elevated for individuals who had the AA type of FAS rs6586161 polymorphism with a high glycemic load. This research recommends appropriate dietary management, specifically avoiding a high-glycemic-load diet, to prevent gastric cancer for people with the AA type in the analysis of rs6586161 polymorphism.

## Figures and Tables

**Table 1:** General characteristics according to gastric cancer cases and controls.

	Cases (*n* = 232)	Controls (*n* = 232)	*p*-Values ^a^
Age (years, mean ± SD)	57.2 ± 11.8	56.6 ± 11.6	0.562
Sex (*n* (%))			
Men	158 (68.1)	158 (68.1)	1.000
Women	74 (31.9)	74 (31.9)	
Body mass index (kg/m^2^, *n* (%))			
Underweight (<18.5)	16 (6.9)	10 (4.3)	0.027
Normal (18.5–22.9)	102 (44.0)	74 (31.9)	
Overweight (23.0–24.9)	46 (19.8)	55 (23.7)	
Obese (≥25.0)	52 (22.4)	75 (32.3)	
Missing	16 (6.9)	18 (7.8)	
Family history of gastric cancer (*n* (%))			
No	201 (86.6)	207 (89.2)	0.393
Yes	31 (13.4)	25 (10.8)	
Hospital (*n* (%))			
Chungnam National University	78 (33.6)	78 (33.6)	1.000
Hanyang University Guri	154 (66.4)	154 (66.4)	
Education (*n* (%))			
≤Elementary school	67 (28.9)	68 (29.3)	0.992
Middle school	33 (14.2)	32 (13.8)	
High school	82 (35.3)	84 (36.2)	
≥College	25 (10.8)	26 (11.2)	
Missing	25 (10.8)	22 (9.5)	
Smoking (*n* (%))			
Never	78 (33.6)	97 (41.8)	0.189
Past	75 (32.3)	67 (28.9)	
Current	79 (34.1)	68 (29.3)	
Alcohol drinking (*n* (%))			
Never	75 (32.3)	76 (32.8)	0.002
Past	45 (19.4)	26 (11.2)	
<20 g/day for women or <40 g/day for men	56 (24.1)	89 (38.4)	
≥20 g/day for women or ≥40 g/day for men	56 (24.1)	41 (17.7)	
*H. pylori* infection (*n* (%))			
Negative	82 (35.3)	58 (25.0)	0.003
Positive	71 (30.6)	105 (45.3)	
Missing	79 (34.1)	69 (29.7)	
FAS rs6586161 polymorphism (*n* (%))			
TT	71 (30.6)	91 (39.2)	0.043
TA	104 (44.8)	103 (44.4)	
AA	57 (24.6)	38 (16.4)	
Total energy intake (kcal/day, mean ± SD)	1854.4 ± 713.9	1703.5 ± 593.4	0.014
Glycemic index (mean ± SD)	58.5 ± 3.4	58.4 ± 3.1	0.597
Glycemic load (mean ± SD)	181.2 ± 68.9	163.2 ± 50.9	0.002
Fruit intake (g/day, mean ± SD)	137.6 ± 165.6	129.8 ± 210.4	0.655
Vegetable intake (g/day, mean ± SD)	60.8 ± 79.3	52.2 ± 51.2	0.169

^a^ *p*-values by Chi-square test for categorical variables or *t*-test for continuous variables.

**Table 2:** Odds ratios (ORs) and 95% confidence intervals (95% CIs) of gastric cancer by general risk factors.

	Cases (*n* = 232)	Controls (*n* = 232)	OR ^a^ (95% CI)
Body mass index (kg/m^2)^			
Underweight (<18.5)	16	10	1.00 (reference)
Normal (18.5–22.9)	102	74	0.80 (0.33–1.93)
Overweight (23.0–24.9)	46	55	0.46 (0.18–1.18)
Obese (≥25.0)	52	75	0.41 (0.17–1.03)
Family history of gastric cancer			
No	201	207	1.00 (reference)
Yes	31	25	1.39 (0.76–2.54)
Education			
≤Elementary school	67	68	1.00 (reference)
Middle school	33	32	1.35 (0.70–2.59)
High school	82	84	1.21 (0.70–2.11)
≥College	25	26	1.46 (0.69–3.09)
Smoking			
Never	78	97	1.00 (reference)
Past	75	67	1.85 (0.96–3.56)
Current	79	68	1.82 (0.98–3.38)
Alcohol drinking			
Never	75	76	1.00 (reference)
Past	45	26	2.15 (1.09–4.23)
<20 g/day for women or <40 g/day for men	56	89	0.71 (0.42–1.21)
≥20 g/day for women or ≥40 g/day for men	56	41	1.58 (0.85–2.93)
*H. pylori* infection			
Negative	82	58	1.00 (reference)
Positive	71	105	0.47 (0.29–0.77)

^a^ Mutually adjusted for sex (men or women), age (years, continuous), body mass index (<18.5, 18.5–22.9, 23.0–24.9, ≥25.0 kg/m^2^, or missing), family history of gastric cancer (no or yes), hospital (Chungnam National University or Hanyang University Guri), education (≤elementary school, middle school, high school, ≥college, or missing), smoking (never, past, or current), alcohol drinking (never, past, <20 g/day for women or <40 g/day for men, or ≥20 g/day for women or ≥40 g/day for men), and *H. pylori* infection (negative, positive, or missing).

**Table 3:** Odds ratios (ORs) and 95% confidence intervals (95% CIs) of gastric cancer by carbohydrate intake, glycemic index, glycemic load, and FAS rs6586161 polymorphism.

	Cases (*n* = 232)	Controls (*n* = 232)	OR ^b^ (95% CI)	OR ^c^ (95% CI)
Carbohydrate ^a^ (g)				
Lowest	68	77	1.00 (reference)	
Medium	71	78	1.01 (0.62–1.66)	
Highest	93	77	1.41 (0.85–2.34)	
Glycemic index				
Lowest	69	77	1.00 (reference)	1.00 (reference)
Medium	77	78	1.24 (0.76–2.03)	1.43 (0.86–2.38)
Highest	86	77	1.44 (0.86–2.42)	1.84 (1.07–3.18) *
Glycemic load				
Lowest	59	77	1.00 (reference)	1.00 (reference)
Medium	63	78	1.05 (0.63–1.77)	1.01 (0.60–1.72)
Highest	110	77	2.34 (1.41–3.87) **	2.14 (1.23–3.75) **
FAS rs6586161 polymorphism				
TT	71	91	1.00 (reference)	
TA	104	103	1.19 (0.76–1.85)	
AA	57	38	1.95 (1.13–3.39) *	
Dominant model				
TT	71	91	1.00 (reference)	
TA+AA	161	141	1.39 (0.92–2.09)	
Recessive model				
TT+TA	175	194	1.00 (reference)	
AA	57	38	1.77 (1.08–2.89) *	

^a^ Energy-adjusted intake. ^b^ Adjusted for sex (men or women), age (years, continuous), body mass index (<18.5, 18.5–22.9, 23.0–24.9, ≥25.0 kg/m^2^, or missing), family history of gastric cancer (no or yes), hospital (Chungnam National University or Hanyang University Guri), education (≤elementary school, middle school, high school, ≥college, or missing), smoking (never, past, or current), alcohol drinking (never, past, <20 g/day for women or <40 g/day for men, or ≥20 g/day for women or ≥40 g/day for men), and *H. pylori* infection (negative, positive, or missing). ^c^ Further adjusted for fruit intake (g/day, continuous), vegetable intake (g/day, continuous), and non-carbohydrate energy intake (g/day, continuous). ** *p* < 0.01, * *p* < 0.05.

**Table 4:** Odds ratios (ORs) and 95% confidence intervals (95% CIs) of gastric cancer by interactions of glycemic load and FAS rs6586161 polymorphism.

	Tertiles of Glycemic Load					
	Lowest		Medium		Highest	
	No. of Cases/Controls	OR ^a^ (95% CI)	No. of Cases/Controls	OR ^a^ (95% CI)	No. of Cases/Controls	OR ^a^ (95% CI)
FAS rs6586161 Polymorphism ^b^						
TT	16/28	1.00 (reference)	20/33	0.95 (0.39–2.36)	35/30	2.34 (0.97–5.65)
TA	29/38	1.21 (0.53–2.80)	31/30	1.74 (0.74–4.08)	44/35	2.19 (0.93–5.13)
AA	14/11	2.40 (0.82–7.01)	12/15	1.16 (0.41–3.29)	31/12	5.53 (2.01–15.21) **
Dominant model ^c^						
TT	16/28	1.00 (reference)	20/33	0.95 (0.39–2.35)	35/30	2.29 (0.95–5.50)
TA+AA	43/49	1.45 (0.66–3.19)	43/45	1.52 (0.69–3.37)	75/47	2.94 (1.31–6.60) **
Recessive model ^c^						
TT+TA	45/66	1.00 (reference)	51/63	1.19 (0.66–2.14)	79/65	2.05 (1.11–3.76) *
AA	14/11	2.12 (0.83–5.44)	12/15	1.04 (0.42–2.59)	31/12	5.00 (2.08–11.99) **

^a^ Adjusted for sex (men or women), age (years, continuous), body mass index (<18.5, 18.5–22.9, 23.0–24.9, ≥25.0 kg/m^2^, or missing), family history of gastric cancer (no or yes), hospital (Chungnam National University or Hanyang University Guri), education (≤elementary school, middle school, high school, ≥college, or missing), smoking (never, past, or current), alcohol drinking (never, past, <20 g/day for women or <40 g/day for men, or ≥20 g/day for women or ≥40 g/day for men), *H. pylori* infection (negative, positive, or missing), fruit intake (g/day, continuous), vegetable intake (g/day, continuous), and non-carbohydrate energy intake (g/day, continuous). ^b^ Interaction was significant (*p* < 0.05). ^c^ Interaction was not significant (*p* ≥ 0.05). ** *p* < 0.01, * *p* < 0.05.

## Data Availability

The data presented in this study are available on request from the corresponding author. The data are not publicly available because of the privacy of the subjects.
